# Anticipatory 50-kHz Precontact Ultrasonic Vocalizations and Sexual Motivation: Characteristic Pattern of Ultrasound Subtypes in an Individual Analyzed Profile

**DOI:** 10.3389/fnbeh.2021.722456

**Published:** 2021-08-20

**Authors:** Wiktor Bogacki-Rychlik, Mateusz Rolf, Michal Bialy

**Affiliations:** Laboratory of Centre for Preclinical Research, Department of Experimental and Clinical Physiology, Medical University of Warsaw, Warsaw, Poland

**Keywords:** 50-kHz ultrasonic vocalizations, anticipatory behavior, male rats, sexual experience, sexual motivation, ultrasound subtypes

## Abstract

We verified the hypothesis of the existence of forms of individual-specific differences in the emission of anticipatory precontact vocalization (PVs) indicating individualization related to sexual experience and motivation in male rats. Long-Evans males were individually placed in a chamber and 50-kHz ultrasounds were recorded during 5-min periods. In experiment 1, PVs were recorded before the introduction of a female in four consecutive sessions during the acquisition of sexual experience. In experiment 2, PVs were analyzed in three groups of sexually experienced males: with the highest, moderate, and the lowest sexual motivation based on previous copulatory activity. In both experiments, the total number of ultrasounds, as well as 14 different specific subtypes, was measured. The ultrasound profiles for each male were created by analyzing the proportions of specific dominant subtypes of so-called 50-kHz calls. We decided that the dominant ultrasounds were those that represented more than 10% of the total recorded signals in a particular session. The number of PVs was positively correlated with the acquisition of sexual experience and previous copulatory efficiency (measured as the number of sessions with ejaculation). Furthermore, PVs showed domination of the frequency modulated signals (complex and composite) as well as flat and short with upward ramp ultrasounds with some individual differences, regardless of the level of sexual motivation. The results show a characteristic pattern of PVs and confirm the hypothesis that the number of PVs is a parameter reflecting the level of sexual motivation.

## Introduction

Ultrasonic vocalization is one of the most intensively studied components of social behavior in rodents. In rats, two main types of ultrasonic vocalization (USV) can be distinguished based on the dominant frequency of the signal: 22-kHz and 50-kHz (Sales and Pye, 1974; Barfield et al., 1979; Brudzynski, 2015, 2021). The first is characterized by long-lasting flat calls (up to 3 s in duration) with a relatively narrow frequency range around 22-kHz (Barfield and Geyer, 1972). In the frustration state (situation of the absence of expected appetitive reinforcement), these calls can be frequency modulated (20–35 kHz) with the preceding element at a frequency of about 45-kHz (Geyer et al., 1978; Bialy et al., 2019b). The so-called 50-kHz USV includes shorter ultrasounds (typically lasting up to several tens of milliseconds) with frequencies from 30–35 to 80-kHz. Signals of such calls, after undergoing Fourier transformations, display various shapes with 14 different subtypes (Wright et al., 2010). The rats emit 50-kHz USV during different elements of behavior related to their high arousal states (Bell, 1974; Berz et al., 2021) such as: socio-sexual interactions including copulation (Barfield et al., 1979; Bialy et al., 2000; Burgdorf et al., 2008), fighting (Sales, 1972; Burke et al., 2017), playing (Reinhold et al., 2019), and even tickling by the experimenter (Burgdorf et al., 2005; Panksepp, 2005). Additionally, pharmacologically induced high levels of general arousal [related to movement activity and sensory sensitization associated with increased level of wakefulness during activation of the gigantocellular reticular nucleus and associated structures (Pfaff, 2017)] result in the expression of 50-kHz vocalizations with a strong positive correlation between the number of 50-kHz ultrasounds emitted and the level of activation of the dopaminergic and noradrenergic pathways (Brudzynski, 2007, 2015; Wright et al., 2012; Simola, 2015; Hamed et al., 2016; Simola and Costa, 2018; Kuchniak et al., 2019).

Contrary to 50-kHz USV and according to the arousal hypothesis, 22-kHz USV occurs during abrupt decreases of arousal (Bell, 1974) and in this case can reflect a relaxation state after ejaculation (Bialy et al., 2016) or a safety signal during aversive conditioning (Jelen et al., 2003).

Although USV is accompanied by certain behavioral situations, the question about the function of 50-kHz vocalizations is still open. One hypothesis assuming a non-semiotic communicative character of vocalization was proposed by Sales and Pye in 1974. An alternative hypothesis postulated that USV can simply be artifacts associated with breathing patterns in rodents (Blumberg and Alberts, 1991). Some researchers have also suggested that it is possible to accurately assess a rat’s emotional state based on the profile of the signals emitted (Brudzynski, 2015; Simola and Granon, 2019).

In this context, the phenomenon of USV can be considered as a complex central response containing an easily measurable autonomic reflex component (objective component) along with a co-occurring mental constituent (subjective component) that is difficult to measure. While the precise division between subjective and objective emotional components is not easy to establish, an accurate behavioral analysis may assess the proportion between them. To categorize some aspects of behavior as a reflection of subjective mental processes, it is necessary to demonstrate individual differences in USV expression. This form of individualization in the case of USV emission was shown in mice (Holy and Guo, 2005; Arriaga and Jarvis, 2013; Asaba et al., 2014; Chabout et al., 2015; Zala et al., 2020).

Moreover, Matsumoto et al. (2016) revealed that rats have the potential ability to discriminate calls emitted by their own vs. conspecific. Based on electrophysiological measurements, authors showed an independent reaction of different groups of neurons in the dorsal amygdala (area of the lateral amygdaloid nucleus) responded specifically to own vs. conspecifics ultrasounds calls.

Furthermore, rats seem to be able to diminish the emission of USV while playing hide-and-seek with humans when hiding themselves which requires being quiet even when having an elevated level of arousal (Reinhold et al., 2019). Such results provide a possible approach for the conscious use of USV by rats.

In sexual behavior, 50-kHz vocalization can be observed during anticipation, during the initiation of copulation, during the copulatory performance, and during the late phase of the postejaculatory interval (Barfield et al., 1979; Bialy et al., 1996, 2019b). Furthermore, the medial preoptic area and the nucleus accumbens—the neuronal circuits important in sexual activity (Hull et al., 2002) are involved in 50-kHz emissions (Fu and Brudzynski, 1994), also during sexual activity (Gao et al., 2019; Karigo et al., 2021), and anticipatory sexual behavior (Bialy et al., 2010). The anticipatory precontact 50-kHz vocalization (PVs) is convenient for the analysis of individual male vocalizations related to general arousal along with sexual motivation (Bialy et al., 2019a). The usefulness of this model derives, from the possibility, to separately analyze the ultrasounds emitted by the male in a conveniently short period (absence of the female provides a single source of USV). It allows describing the factors that trigger and modify individual vocalization in an appetitive state. Furthermore, it can also be a convenient parameter in the experimental models based on the acquisition of socio-sexual experience and extinction reactions, facilitate recognition of crucial cues regulating acquisition/extinction reactions. It is due to the direct relationship of PVs with the memory of emotional state, copulatory efficiency, and reward value of preceding socio-sexual interactions (Bialy et al., 2000).

However, this relationship concerns the total number of emitted ultrasounds, while the question of the relationship between the level of sexual motivation and emitted subtypes remains unknown. In the present experiments, we recorded PVs emitted during the acquisition of sexual experience as well as those emitted by sexually experienced males with different levels of copulatory activity. We have classified 50-kHz calls into one of 14 subtypes (Wright et al., 2010) and created individual USV profiles for each male. We compared the profiles thus obtained with the copulatory history of each rat. We would answer whether PVs subtypes profiles have the traits suggesting the uniqueness for each animal. If they have, is there any relation between this emerging inter-individual differentiation and the level of sexual motivation for each animal?

The purpose of this study was to answer the question: are there individual-specific distinctions of 50-kHz vocalization associated with the level of sexual experience and sexual motivation in the ultrasound recordings? If not, what is the general physiological pattern of anticipatory precontact vocalizations in the sexual behavior of the male rat?

## Materials and Methods

### Animals

Long-Evans rats, 6–7 months old, were the subjects in this study.

The choice of animals’ age was dictated by the stabilized profile of sexual parameters occurring between 150 and 500 days of their life (allows to omit the first life period with significant fluctuation in levels of sex hormones and corresponding tissue sensitivity) (Larsson, 1956, 1967).

Males and females were housed in separate rooms with a reversed 12-h light-dark cycle (lights switched off at 09:00 h) and at a temperature maintained at 22 ± 1°C. All of the animals had food and water freely available. The food consisted of standard laboratory chow (experiment 1 and 2) with some enrichment in experiment 2 (cereals, fresh vegetables, and fruits) due to the effort to achieve more natural-like conditions conducive to the emergence of social hierarchies. The males arrived from the Department of Experimental Medicine, Medical University of Silesia, Katowice, from different cohorts for experiments 1 and 2. The males were sexually naïve at the beginning of the experiments and have been described separately in the corresponding experiments.

The females (*N* = 20, 10 in the first and 10 in the second experiment), were housed 2–3 animals per standard laboratory cage (55.6 cm × 33.4 cm × 19.5 cm).

The housing conditions of males are described in corresponding “experiment 1 and 2” subsections.

All of the cages were provided with wood shavings and dedicated plastic tubes as enrichment. The ovariectomized females were brought into estrus with a single injection of estradiol benzoate (50 μg/rat s.c., Sigma-Aldrich) and progesterone (500 μg/rat s.c., Sigma-Aldrich). Hormones were dissolved in sesame oil and administered at a dose of 0.05 ml per individual. Hormonal injections were given 48–72 h before the test for estradiol and 4–8 h before the test for progesterone. Estrus was induced not more often than once a week and not less often than once every 2 weeks. The females were sexually experienced at the beginning of experiments. During an experimental day, a female copulated with up to two males.

### Behavioral Procedures

All of the behavioral tests were conducted between 13:00 and 17:00 h during the dark phase of the light-dark cycle. We maintained at least a 1-week interval between tests to counteract the influence of the sexual exhaustion phenomenon on the sexual parameters, which is particularly important in less active groups of males (Larsson, 1956).

The test chamber was a transparent Plexiglas container (50 cm × 25 cm × 30 cm) in experiment 1 and a container made of transparent reinforced polyethylene (39 cm × 59 cm × 37 cm) in experiment 2.

Before an experiment, all of the males were acclimated 3–4 times to the experimental chamber for 10 min the first time and then for 5 min in consecutive acclimating sessions.

### Acquisition of Sexual Experience

A male was introduced into the experimental chamber and a female was introduced 5 min later. Ultrasounds were recorded during the 5-min period between the introduction of the male and the female to the experimental chamber (precontact anticipatory ultrasonic vocalizations—PVs). The session was conducted until the first ejaculation and ended just after the male resumed copulatory activity. The maximum duration of a single session was 30 min and the session was terminated after this time.

### Ultrasound Analysis and Behavior Recording

Visual recording of behavior was made using the Noldus EthoVision system.

Simultaneous to visual recording, ultrasounds were recorded on the same computer using the Metris Sonotrack system.

The microphone was placed 50 cm above the floor during ultrasonic recording. Spectral analysis of ultrasounds was performed using the Sonotrack software. Each ultrasound was analyzed manually using the Sonotrack cursor and was assigned to one of the 14 subtypes according to the classification proposed by Wright et al. (2010) based on its characteristic shape, complexity, and average frequency.

Each session was independently analyzed by two experienced observers to minimize bias in classification. The most inconclusive results were found between the complex and composite categories. For this reason, we decided to modify the classification and distinguish only 13 subtypes. Therefore, the complex and the composite subtypes were combined into one complex/composite category.

In this classification, subtypes of ultrasounds are defined as: complex/composite, trill, flat-trill, trill with jumps, split, step up, step down, multi-step, flat, short, upward ramp, downward ramp, and inverted U. In ambiguous cases, the ultrasounds were listened to at slow speed to classify them correctly.

Subsequently, we counted the total number of ultrasounds and the proportion of their subtypes emitted by each male. The identification of dominant subtypes in the recording was used to create a simplified code characteristic for each individual. Subtypes accounting for at least 10% of the total number of signals were understood to be dominant. Additionally, a low proportion (less than 10%) of the flat vocalization, which is usually frequently emitted by male rats, was considered as a distinguishing feature. In experiment 1, a characteristic vocalization profile for each male was created based on the dominant subtypes visible in all sessions with vocalizations above 9 USV.

### Experiment 1

Males (*N* = 17) were housed 2–3 animals per standard laboratory cage (55.6 cm × 33.4 cm × 19.5 cm). All were sexually naïve at the beginning of the experiment. They acquired sexual experience during four consecutive copulatory sessions. Our previous results showed that during four sessions changes in sexual parameters related to the acquisition of sexual experience are most relevant and stabilized around the fifth session (Bialy et al., 2000).

All of the precontact 50-kHz vocalizations (PVs), quantification of every subtype and their percentage share in the total spectrum were analyzed. The sum of PVs was additionally correlated with copulatory efficiency measured as the sum of sessions when the male achieved ejaculation (during sessions 2–4 after the male had his first sexual experience). One male was excluded from the experiment due to extremely aggressive behavior.

### Experiment 2

Males (*N* = 20) were housed in the enriched environment as the group contained up to five animals which supported hierarchization within the group. These special larger cages had a vertical structure with wire sidewalls and a solid base (base dimensions 48 cm × 80 cm and 142 cm high) and were equipped with three levels of wooden platforms. The males before the experimental test achieved socio-sexual experience during five copulatory training sessions.

After such training, rats achieved a relatively stable level of sexual performance related to sexual experience (Bialy et al., 2000). During each session, the male was placed individually with a receptive female in one cage. Training sessions were terminated after 30 min. As the indicator of copulatory efficiency, we used the number of sessions when males achieved ejaculation(s) (or lack of them) from all five training sessions. Then we divided each male into one of three groups based on their copulatory history.

Alpha males (*N* = 8) ejaculated at least during two sessions, beta males (*N* = 7) ejaculated only once and gamma males (*N* = 5) never ejaculated during the five copulatory sessions.

One week after the last training session, precontact anticipatory ultrasonic vocalizations were recorded. A male was introduced for 5 min into the familiar experimental/training chamber where odor cues from receptive females were present. The scent stimuli were provided by the earlier placement of three randomly selected receptive females for 5 min into the experimental cage. Prior to the introduction of the male, the females were taken out.

Males that emitted less than 10 ultrasounds were excluded from statistical analysis. This criterion was adopted because, in the case of few ultrasounds, a single vocalization showed a disproportionately high percentage of a given subtype of vocalization, which led to a significant error in the interpretation of the results. This criterion was met by a total of three males—one male from each examined group.

### Statistics

Data from the acquisition phase (the number of ultrasounds, mount, intromission, and ejaculation latencies) were analyzed by non-parametric Friedman repeated measure ANOVA and Dunn’s *post hoc* tests (Experiment 1). The number of ultrasounds in Experiment 2 was analyzed by non-parametric Kruskal–Wallis ANOVA and Mann–Whitney for independent groups. Additionally, in experiment 1, the Spearman test was used to correlate the number of the session with ejaculation(s) with the total number of PVs.

## Results

### Experiment 1

#### Number of PVs

**FIGURE 1:**
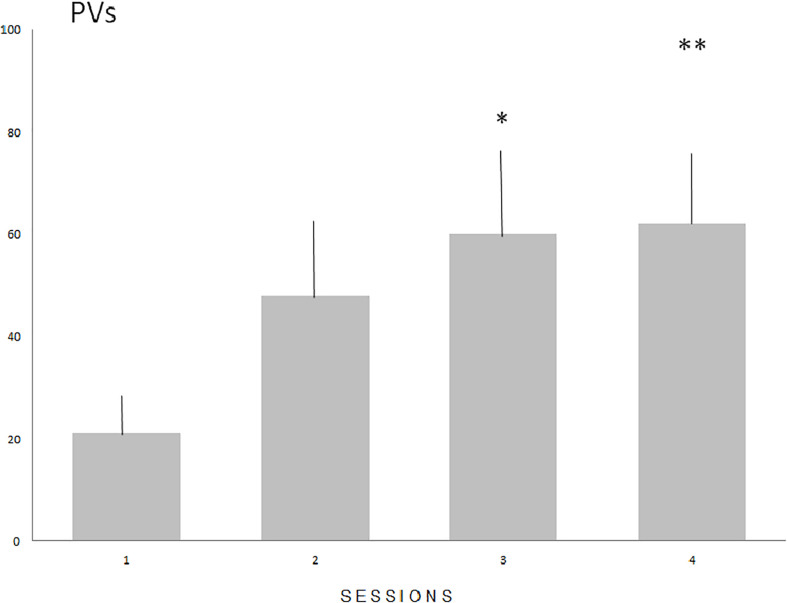
The means number (SE) of PVs during the acquisition of sexual experience. Numbers on the Y axis represent the means value of PVs emitted during subsequent sessions. Statistically significant differences compared with session 1 (^∗^*P* < 0.05 and ^∗∗^*P* < 0.01).

The number of PVs (Figure 1) increased significantly in males (*N* = 16) during four consecutive sessions of the acquisition of sexual experience (Fr = 12.816, *P* < 0.01 with statistically significant differences between sessions 1 and 3 (*P* < 0.05), and sessions 1 and 4 (*P* < 0.01). Mount latency decreased significantly (Fr = 17.591, *P* < 0.001) with statistically significant differences between sessions 1 and 2 (*P* < 0.05), sessions 1 and 3 (*P* < 0.01) and 1 and 4 (*P* < 0.01. Intromission and ejaculation latencies display no significant differences (*P* = 0.0815 and *P* = 0.077 respectively).

**FIGURE 2:**
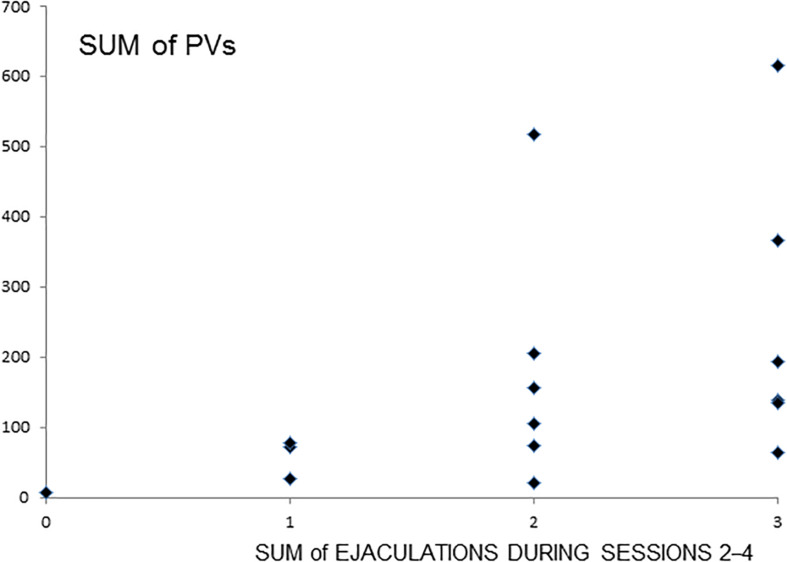
Total number of PVs with relation to the sum of ejaculations during sessions 2–4. Numbers on the X-axis represent the sum of ejaculation from 0 to 3 achieved by the male during all three sessions 2–4. 0- means that male did not achieve any ejaculation during three sessions, respectively, 1—only one ejaculation during three sessions, 2—two ejaculations, and 3—ejaculated in each session. Numbers on the Y-axis represent the sum of PVs emitted by the male during sessions 2–4. The diamond represents an individual male. *r* = 0.5608, *P* < 0.05.

A significant correlation (Spearman *r* = 0.5608, *P* < 0.05, *N* = 16) was found between the number of sessions with ejaculation (sessions 2–4) and the total number of PVs emitted by a male during these sessions (Figure 2).

#### PVs Subtypes

We counted the percentage of different subtypes when a male vocalized 10 times or more in a session. An analysis of USV subtypes was performed and more than 10 vocalizations occurred the most often in three sessions for the same male (six males) and less frequently: in four sessions (five males), in two sessions (four males), and one male vocalized less than 10 times in all four sessions. Those subtypes that appeared at least 10% in the total pool of male vocalizations were marked as the dominant calls. Table 1 shows those subtypes that predominated in all sessions (100%) and those that occurred in at least two sessions out of three or four (50–75%) sessions and in only one session (25%). The complex/composite and flat (or short or upward ramp) subtypes occurred most frequently. The complex/composite and flat (or short or upward ramp) profile was typical to anticipatory precontact vocalizations regardless of the male’s copulatory efficiency as measured by the number of ejaculations achieved during sessions 2–4.

**TABLE 1:** PVs and ultrasonic subtypes during consecutive sessions.

#	Copulatory status	100%	50–75%	25%	Note
1	3	Sh, UR	F, SU, CC	DR, IU	
2	3	CC, F, Sh	UR	Trills	
3	3	CC, Sh	UR	SU, Trills	Rare flat
4	3	UR, F, CC	Sh, SU	/	
5	3	F, Sh, SD, CC	/	/	
6	3	CC	Trills, UR, Sh	F	
7	2	UR, Sh, F	SD, CC, SU	/	
8	2	CC	Trills, SU, MS	SD, UR, Sh, IU	Rare flat
9	2	CC, Sh, UR	Trills, SU	/	Rare flat
10	2	F, CC	SD, Sh	SU,UR, Trills	
11	2	Trills, CC	/	F, Sh	
12	2	CC, F	/	Trills, SD, Sh	
13	2	F, CC	/	Trills, Sh, UR	
14	1	CC	Sh, UR, Trills, F	MS, SU	
15	1	CC, UR, Trills	/	/	Rare flat
16	0	/	/	/	

*Subtypes of ultrasounds are defined as: complex and composite (CC), trill, flat-trill, and trill with jumps (Trills), split (S), step up (SU), step down (SD), multi-step (MS), flat (F), short (Sh), upward ramp (UR), downward ramp (DR), and inverted U (IU). The columns show subtypes emitted always (100%), frequently (50–75%), and sporadically (25%). Within the columns, the order from left to right indicates a decreasing percentage of emitted subtypes.*

**FIGURE 3:**
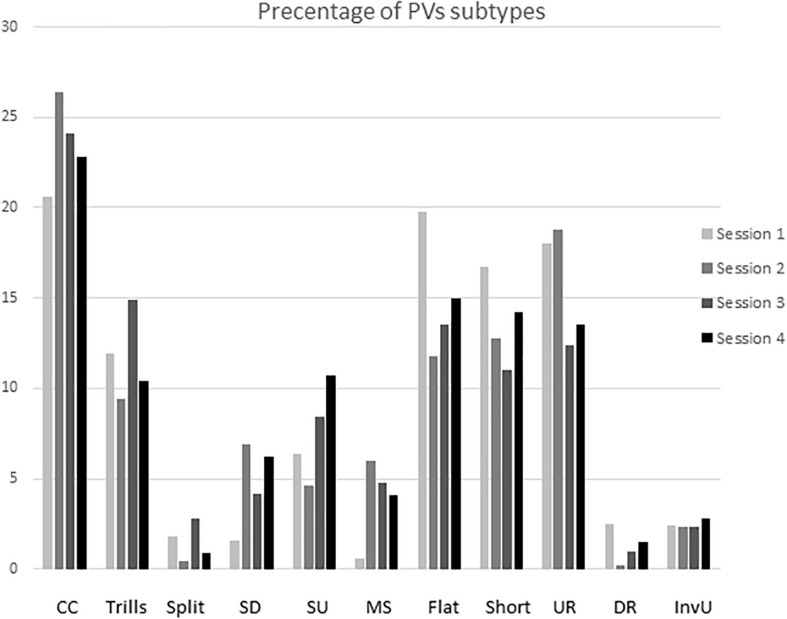
Means percentage of different ultrasonic subtypes during the acquisition of sexual experience. Subtypes are represent as: complex and composite (CC), trill, flat-trill and trill with jumps (Trills), split, step up (SU), step down (SD), multi-step (MS), flat, short, upward ramp (UR), downward ramp (DR), and inverted U (IU). Subtypes on the X-axis are shown from left to right of each column as sessions 1, 2, 3, and 4.

Subtypes of PVs did not differ as a function of the acquisition of sexual experience. The percentage of different subtypes showed a similar level during all four copulatory sessions (Figure 3).

### Experiment 2

#### Number and Subtypes of PVs

**FIGURE 4:**
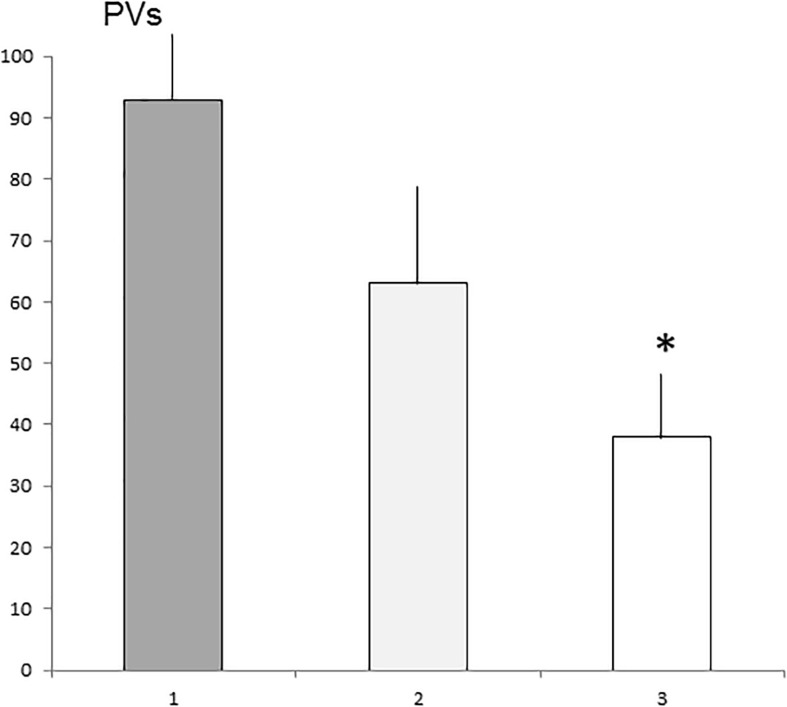
The means number (SE) of PVs emitted by alpha, beta, and gamma groups. 1—Alpha group, 2—Beta group, and 3—Gamma group. ^∗^Statistically significant differences (*P* < 0.05) compared with the alpha group.

The number of PVs differed significantly between the alpha, beta, and gamma groups [H(2), *N* = 17] = 6.0396, *P* < 0.05. The alpha group vocalized more frequently compared with the gamma group (*P* < 0.05). The beta group did not differ significantly compared with the alpha and gamma groups (Figure 4).

The three most frequent subtypes of ultrasounds detected were: complex/composite (alpha 39.9%, beta 42.4%, and gamma 38% of total ultrasounds), flat (alpha 23.1%, beta 23.8%, gamma 24% of total ultrasounds) and upward ramp (alpha 10.2%, beta 11.5%, gamma 9.2% of total ultrasounds).

**FIGURE 5:**
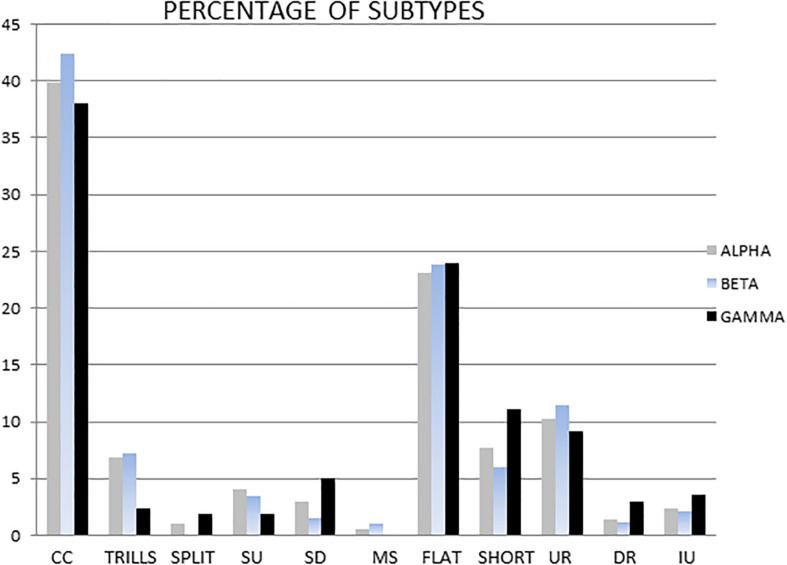
The dominant means percentage of different subtypes in the alpha, beta and gamma groups. Subtypes are shown from left to right as: complex and composite (CC), trill, flat-trill and trill with jumps (Trills), split, step up (SU), step down (SD), multi-step (MS), flat, short, upward ramp (UR), downward ramp (DR), and inverted U (IU).

There were no statistically significant differences in the percentage of selected subtypes in the total pool of ultrasounds emitted by males when these three groups were compared (Figure 5). Table 2 shows the main ultrasonic subtypes emitted by each male.

**TABLE 2:** The individual profiles of emitted ultrasounds.

Male number	Group	Pattern
1	Alpha	CC, Sh, -F
2	Alpha	CC, F
3	Alpha	CC, F
4	Alpha	F, CC
5	Alpha	CC, F, UR
6	Alpha	CC, F, Sh, UR
7	Alpha	CC, F, UR
8	Beta	F,CC
9	Beta	CC, UR, F
10	Beta	CC, T -F
11	Beta	CC -F
12	Beta	CC, UR, F
13	Beta	F, CC
14	Gamma	F, CC, UR, SD
15	Gamma	F, CC
16	Gamma	CC, UR, -F
17	Gamma	CC, Sh, F

*In columns, the order from left to right indicates decreasing percentage. Individual vocalization patterns based on the dominant subtypes of individual males appear in the last column of the table. The rare occurrence of the flat subtype is also considered as a minus F. Subtypes of ultrasounds are defined as: complex and composite (CC), trills (T), step down (SD), multi-step (MS), flat (F), short (Sh), upward ramp (UR), downward ramp (DR), and inverted U (IU).*

## Discussion

The results indicate a significant positive relationship between the total number of anticipatory precontact vocalizations and the number of sessions with achieved ejaculation(s). It reveals an association between the level of socio-sexual motivation and the expression of PVs. Shortening of mount latency—parameter related to sexual motivation (Hull et al., 2002) during the acquisition of sexual experience additionally supports the relation between PVs and sexual motivation.

In two independent groups of rats (experiments 1 and 2), similar patterns of emitted ultrasounds were observed. In all of the males, regardless of their level of sexual motivation or sexual experience, the dominant signals were the frequency modulated complex/composite (CC) signals and the unmodulated flat (F) and, less frequently, the slightly modulated short (Sh) and upward ramp (UR) ultrasounds appeared as co-occurring subtypes. Trills were emitted on an elevated level in experiment 1 only. Other signals were seen sporadically and rarely exceeded 10% of the total PVs.

### PVs and Sexual Activity

Anticipatory precontact vocalizations depend on the level of sexual activity and reward value of the contacts. In experiment 1, the number of PVs increased during the acquisition of sexual experience.

Moreover, the total number of PVs relates to the number of ejaculations. Ejaculation has the highest reward value compared with other elements of copulatory behavior (Tenk et al., 2009) and, in this context, PVs are related to the reward value of socio-sexual contacts. In experiment 2, the active males vocalized on a significantly higher level compared with much less active or sexually inactive males.

A more detailed explanation of the processes related to anticipatory ultrasonic precontact vocalizations follows. The number of PVs depends on the acquisition of sexual experience, conditioning to odor and background cues, the reward value of contact, NMDA (Bialy et al., 2000), and D1 receptor activity (Bialy et al., 2010).

Blocking of the NMDA receptor (receptor important in neuronal plasticity processes) inhibited changes in PVs during the acquisition of sexual experience but have no significant effect on PVs in sexually experienced males (Bialy et al., 2000). Acquisition of sexual experience and PVs are also related to D1 receptor activity. Surprisingly, peripheral repeated administration of both: antagonist and agonist of D1 receptor inhibited changes in PVs during the acquisition phase (Bialy et al., 2010) but, D1 receptor agonist had a minor effect on PVs in sexually experienced males (Beck et al., 2002). Repeated D1 receptor agonist injections into the nucleus accumbens similarly diminished PVs during the acquisition phase and this effect prolonged at least 4 weeks.

Previously, were identified the specific, dopaminergic neuronal group in the nucleus accumbens responding either to appetitive unconditioned and conditioned stimuli that regulating, reward related behavior (Fiorillo et al., 2003). Thus, probably any significant disturbance of their activity: inhibition or overstimulation can be responsible for such attenuation of the acquisition phase and diminishing PVs.

In this context, PVs reflect learning and emotional memory processes. The number of PVs seems to be related to the level of general arousal and sexual motivation rather than to sexual arousal (Bialy et al., 2019a). In addition, social motivation should also be taken into account, as contacts with an anestrus female also has a triggering effect on PVs although at a significantly lower level (Bialy et al., 2000). We can assume that in the present experiments, PVs in low sexually active males were related to social motivation and general arousal rather than sexual motivation. All of these data seem to be consistent and repeatable, enabling the changing number of PVs to be used as a parameter for the functioning of the memory circuits related to the reward system.

### PVs and Individualization

We observed individual differences in the percentage of non-dominant (less than 10% of the total spectrum) subtypes as well as in the occasionally very low level of the flat subtype. The composite signals contained within the CC group consisted of a modulated sound of the complex subtype with a directly accompanying sound of the short subtype. The distinction between the two ultrasounds was often only possible after listening to the signal. In some studies, this type of signal is listed as a “dual-type.” However, due to the high subjectivity in the evaluation between different observers and the inability to listen on different recording systems other than our system, we decided on a common category. The composite subtype calls, consisting of calls other than complex and short components, occurred very rarely. Furthermore, the individual specificity of vocalizations was manifested in different proportions in the short, upward ramp, and flat subtypes. In general, the presented form of individualization seems to be subtle and not easy to use as a behavioral parameter.

This limitation is also related to the fact that a vast majority of inter-individual differentiation can occur within the so-called frequency-modulated non-trill group (split, step down, step up, multistep, downward, and inverted U) Despite their lack of visibility in the profiles after applying our quantitative criterion, variation within these types exists. Each of them is characterized by its specific shape, although their acoustic parameters, such as duration, mean frequencies, or modulation ranges, are heterogeneous. In summary, the observed individualization additionally occurs within the types of ultrasound that occur sporadically, in their exact parameters, and their potential combinations (like syllables).

Nevertheless, given the existence of the presented pattern, we can assume that it reflects a relatively constant emotional state. In this context, large, comparatively easy to visualize deviations from it may indicate significant central changes. The question of a behaviorally relevant reason for the existence of certain forms of individualization remains open.

### USV Profiles

The main question, and aim of our experiments, was to answer the question: do changes in the level of PVs related to elevated general arousal and sexual motivation correspond to changes in the profile of the ultrasound subtypes. Qualitative analysis of ultrasound subtypes revealed an existing characteristic vocalization pattern with complex/composite subtypes combined with flat, short, and upward ramp subtypes dominating in the recordings. This model of vocalization appears similar in both sexually inactive and active males. Moreover, it seems constant during consecutive sessions of sexual experience acquisition and sexual motivation influenced the number of PVs but not the subtype profile. In experiment 2, more escalated social hierarchization was evident in the form of an increase in the non-copulatory number of rats (the gamma group). Regardless of this, the pattern of the profiles remained similar.

In our experiments, trills were visible in some sexually moderately active males. They did not change as a function of sexual experience. Probably they are related to the general arousal level but not sexual motivation *per se*. This hypothesis can be supported by the results of experiments with the repeated administration of psychostimulant drug agents (amphetamine and derivatives, apomorphine, cocaine) which evokes the expression of the trills subtype (Wright et al., 2012, 2013; Mulvihill and Brudzynski, 2018). However, the biological context of the expression of this type of vocalization is not entirely clear. For example, Mahler et al. (2013) observed no difference in the number of trill emissions during the methamphetamine self-administration period and the subsequent extinction period, obtaining an increase only after re-exposure of the animals to the drug.

Furthermore, many authors, due to the simplicity of ultrasonic classification, often cluster all frequency modulated subtypes (Taracha et al., 2012; Brenes and Schwarting, 2015; Mulvihill and Brudzynski, 2018). The lack of correlation between sexual motivation and the profile of PVs could indicate that the specific type of frequency modulated calls presented in our experiments is an innate pattern of general anticipatory activity. In this context, the degree of modulation could represent a continuous behavioral spectrum, with complex/composite subtype domination associated with more physiological situations and the trills subtype domination reflecting highly aroused states. Further research is needed to confirm this hypothesis. Also, the neurobehavioral context of flat, unmodulated subtype is not clear. In these experiments, we observed flat call as one of the dominant types in most rats. However, in few animals, we observed a noticeable decrease of flats calls in profiles (Tables 1, 2). This reduction wasn’t related to copulatory status (activity), indicating the involvement of a factor other than socio-sexual motivation. Recently, some authors proposed that this type can be associated with social-coordinating function and alimentary appetitive reactions, including the food approach (Brudzynski, 2021). According to our results, additional data are needed to clarify this issue.

### Further Implications

We have shown that the pattern of physiological anticipatory vocalization was relatively constant and persisted from one experimental session to the next. It is worth noting, however, that vocalization can change significantly in some situations. We observed rapid changes in vocalization occurring during a single session with characteristic transitions between the 50-kHz and 22-kHz frequencies during the frustrated states (Bialy et al., 2019b). Using an analogous non-contact model, changes in the vocalization profile across consecutive sessions have also been demonstrated in mice (Zala et al., 2020). Similarly, significant changes during consecutive experimental sessions have been shown repeatedly with addiction models, e.g., morphine (Covington and Miczek, 2003; Hamed and Kursa, 2018), cocaine (Ma et al., 2010), and methamphetamine (Mahler et al., 2013). Another example of such an application is the change in vocalization profiles that have been observed in induced Parkinson’s disease in rats where ultrasound flattening occurs as the disease progresses (Ciucci et al., 2009). Individual vocalization and own vs. foreign recognition can be disturbed in schizophrenic-like symptoms in the rat model (Matsumoto et al., 2016). Furthermore, changes in the vocalization profile occur in the rat model of affective disorders and psychotic states (Nikiforuk et al., 2013; Wendler et al., 2019; Wöhr, 2021) as well as in suggested models of autism (Caruso et al., 2020).

## Conclusion

We proposed a relatively simple method to discriminate individual characters of ultrasonic vocalization in rats based on dominant subtype ultrasonic vocalizations. Our results indicate a persistent similar vocalization pattern during anticipatory behavior, regardless of the level of socio-sexual motivation or experience. The number of ultrasonic vocalizations but not the different subtypes seems to be the most related to sexual motivation.

## Data Availability Statement

The raw data supporting the conclusions of this article will be made available by the authors, without undue reservation.

## Ethics Statement

The animal study was reviewed and approved by First Local Ethical Committee in Warsaw.

## Author Contributions

WB-R and MB designed and performed experiments, analyzed data, and prepared the manuscript. MR analyzed USV data in experiments 2. All authors contributed to the article and approved the submitted version.

## Conflict of Interest

The authors declare that the research was conducted in the absence of any commercial or financial relationships that could be construed as a potential conflict of interest.

## Publisher’s Note

All claims expressed in this article are solely those of the authors and do not necessarily represent those of their affiliated organizations, or those of the publisher, the editors and the reviewers. Any product that may be evaluated in this article, or claim that may be made by its manufacturer, is not guaranteed or endorsed by the publisher.

## References

[B1] Arriaga G. Jarvis E. D. (2013). Mouse vocal communication system: are ultrasounds learned or innate? *Brain Lang.* 124 96–116. 10.1016/j.bandl.2012.10.002 23295209 PMC3886250

[B2] Asaba A. Hattori T. Mogi K. Kikusui T. (2014). Sexual attractiveness of male chemicals and vocalizations in mice. *Front. Neurosci.* 8:231. 10.3389/fnins.2014.00231 25140125 PMC4122165

[B3] Barfield R. J. Geyer L. A. (1972). Sexual behavior: ultrasonic postejaculatory song of the male rat. *Science* 176 1349–1350. 10.1126/science.176.4041.1349 5034552

[B4] Barfield R. J. Auerbach P. Geyer L. A. McIntosh T. K. (1979). Ultrasonic Vocalizations in Rat Sexual Behavior. *Am. Zool.* 19 469–480. 10.1093/icb/19.2.469 31919651

[B5] Beck J. Biały M. Kostowski W. (2002). Effects of D(1) receptor agonist SKF 38393 on male rat sexual behavior and postcopulatory departure in the goal compartment-runway paradigm. *Physiol. Behav.* 76 91–97. 10.1016/s0031-9384(02)00678-9 12175592

[B6] Bell R. W. (1974). Ultrasounds in small rodents: arousal-produced and arousal-producing. *Dev. Psychobiol.* 7 39–42. 10.1002/dev.420070107 4855837

[B7] Berz A. Pasquini, de Souza C. Wöhr M. Schwarting R. K. W. (2021). Limited generalizability, pharmacological modulation, and state-dependency of habituation towards pro-social 50-kHz calls in rats. *Science* 24:102426. 10.1016/j.isci.2021.102426 33997703 PMC8102916

[B8] Bialy M. Beck J. Abramczyk P. Trzebskj A. Przybylski J. (1996). Sexual behavior in male rats after nitric oxide synthesis inhibition. *Physiol. Behav.* 60 139–143. 10.1016/0031-9384(95)02272-4 8804654

[B9] Bialy M. Bogacki-Rychlik W. Kasarello K. Nikolaev E. Sajdel-Sulkowska E. M. (2016). Modulation of 22-khz postejaculatory vocalizations by conditioning to new place: Evidence for expression of a positive emotional state. *Behav. Neurosci.* 130 415–421. 10.1037/bne0000153 27454624

[B10] Bialy M. Bogacki-Rychlik W. Przybylski J. Zera T. (2019a). The Sexual Motivation of Male Rats as a Tool in Animal Models of Human Health Disorders. *Front. Behav. Neurosci.* 13:257. 10.3389/fnbeh.2019.00257 31956302 PMC6947634

[B11] Bialy M. Kalata U. Nikolaev-Diak A. Nikolaev E. (2010). D1 receptors involved in the acquisition of sexual experience in male rats. *Behav. Brain Res.* 206 166–176. 10.1016/j.bbr.2009.09.008 19747509

[B12] Bialy M. Podobinska M. Barski J. Bogacki-Rychlik W. Sajdel-Sulkowska E. M. (2019b). Distinct classes of low frequency ultrasonic vocalizations in rats during sexual interactions relate to different emotional states. *Acta Neurobiol. Exp.* 79 1–12. 10.21307/ane-2019-001 31038481

[B13] Bialy M. Rydz M. Kaczmarek L. (2000). Precontact 50-kHz vocalizations in male rats during acquisition of sexual experience. *Behav. Neurosci.* 114 983–990. 10.1037/0735-7044.114.5.983 11085613

[B14] Blumberg M. S. Alberts J. R. (1991). On the significance of similarities between ultrasonic vocalizations on infant and adult rats. *Neuroscie. Biobehav. Rev.* 15 383–390. 10.1016/S0149-7634(05)80031-4 1956606

[B15] Brenes J. C. Schwarting R. K. W. (2015). Individual differences in anticipatory activity to food rewards predict cue-induced appetitive 50-kHz calls in rats. *Physiol. Behav.* 149 107–118. 10.1016/j.physbeh.2015.05.012 25992480

[B16] Brudzynski S. M. (2007). Ultrasonic calls of rats as indicator variables of negative or positive states: acetylcholine-dopamine interaction and acoustic coding. *Behav. Brain Res.* 182 261–273. 10.1016/j.bbr.2007.03.004 17467067

[B17] Brudzynski S. M. (2015). Pharmacology of Ultrasonic Vocalizations in adult Rats: Significance, Call Classification and Neural Substrate. *Curr. Neuropharmacol.* 13 180–192. 10.2174/1570159x13999150210141444 26411761 PMC4598430

[B18] Brudzynski S. M. (2021). Biological Functions of Rat Ultrasonic Vocalizations, Arousal Mechanisms, and Call Initiation. *Brain Sci.* 11:605. 10.3390/brainsci11050605 34065107 PMC8150717

[B19] Burgdorf J. Kroes R. A. Moskal J. R. Pfaus J. G. Brudzynski S. M. Panksepp J. (2008). Ultrasonic vocalizations of rats (Rattus norvegicus) during mating, play, and aggression: Behavioral concomitants, relationship to reward, and self-administration of playback. *J. Comp. Psychol.* 122 357–367. 10.1037/a0012889 19014259

[B20] Burgdorf J. Panksepp J. Brudzynski S. M. Kroes R. Moskal J. R. (2005). Breeding for 50-kHz positive affective vocalization in rats. *Behav. Genet.* 35 67–72. 10.1007/s10519-004-0856-5 15674533

[B21] Burke C. J. Kisko T. M. Pellis S. M. Euston D. R. (2017). Avoiding escalation from play to aggression in adult male rats: The role of ultrasonic calls. *Behav. Process.* 144 72–81. 10.1016/j.beproc.2017.09.014 28941795

[B22] Caruso A. Ricceri L. Scattoni M. L. (2020). Ultrasonic vocalizations as a fundamental tool for early and adult behavioral phenotyping of Autism Spectrum Disorder rodent models. *Neurosci. Biobehav. Rev.* 116 31–43. 10.1016/j.neubiorev.2020.06.011 32544538

[B23] Chabout J. Sarkar A. Dunson D. B. Jarvis E. D. (2015). Male mice song syntax depends on social contexts and influences female preferences. *Front. Behav. Neurosci.* 9:76. 10.3389/fnbeh.2015.00076 25883559 PMC4383150

[B24] Ciucci M. R. Ahrens A. M. Ma S. T. Kane J. R. Windham E. B. Woodlee M. T. (2009). Reduction of dopamine synaptic activity: degradation of 50-kHz ultrasonic vocalization in rats. *Behav. Neurosci.* 123 328–336. 10.1037/a0014593 19331456 PMC2737695

[B25] Covington H. E. Miczek K. A. (2003). Vocalizations during withdrawal from opiates and cocaine: possible expressions of affective distress. *Eur. J. Pharmacol.* 467 1–13. 10.1016/S0014-2999(03)01558-9 12706449

[B26] Fiorillo C. D. Tobler P. N. Schultz W. (2003). Discrete coding of reward probability and uncertainty by dopamine neurons. *Science* 299 1898–1902. 10.1126/science.1077349 12649484

[B27] Fu X. W. Brudzynski S. M. (1994). High-frequency ultrasonic vocalization induced by intracerebral glutamate in rats. *Pharmacol. Biochem. Behav.* 49 835–841. 10.1016/0091-3057(94)90231-3 7886095

[B28] Gao S. C. Wei Y. C. Wang S. R. Xu X. H. (2019). Medial Preoptic Area Modulates Courtship Ultrasonic Vocalization in Adult Male Mice. *Neurosci. Bull.* 35 697–708. 10.1007/s12264-019-00365-w 30900143 PMC6616611

[B29] Geyer L. A. Barfield R. J. McIntosh T. K. (1978). Influence of gonadal hormones and sexual behavior on ultrasonic vocalization in rats: II. Treatment of males. *J. Comparat. Physiol. Psychol.* 92 447–456. 10.1037/h0077487 681563

[B30] Hamed A. Kursa M. B. (2018). Inter-individual differences in serotonin and glutamate co-transmission reflect differentiation in context-induced conditioned 50-kHz USVs response after morphine withdrawal. *Brain Struct. Funct.* 223 3149–3167. 10.1007/s00429-018-1683-4 29774428 PMC6132671

[B31] Hamed A. Daszczuk P. Kursa M. B. Turzyńska D. Sobolewska A. Lehner M. (2016). Non-parametric analysis of neurochemical effects and Arc expression in amphetamine-induced 50-kHz ultrasonic vocalization. *Behav. Brain Res.* 312 174–185. 10.1016/j.bbr.2016.05.042 27288591

[B32] Holy T. E. Guo Z. (2005). Ultrasonic Songs of Male Mice. *PLoS Biol.* 3:e386. 10.1371/journal.pbio.0030386 16248680 PMC1275525

[B33] Hull E. M. Meisel R. L. Sachs B. D. (2002). “1 - Male Sexual Behavior,” in *Hormones, Brain and Behavior*, eds Pfaff D. W. Arnold A. P. Fahrbach S. E. Etgen A. M. Rubin R. T. (San Diego: Academic Press), 3–137.

[B34] Jelen P. Soltysik S. Zagrodzka J. (2003). 22-kHz ultrasonic vocalization in rats as an index of anxiety but not fear: behavioral and pharmacological modulation of affective state. *Behav. Brain Res.* 141 63–72. 10.1016/s0166-4328(02)00321-2 12672560

[B35] Karigo T. Kennedy A. Yang B. Liu M. Tai D. Wahle I. A. (2021). Distinct hypothalamic control of same- and opposite-sex mounting behaviour in mice. *Nature* 589 258–263. 10.1038/s41586-020-2995-0 33268894 PMC7899581

[B36] Kuchniak K. Wyszogrodzka E. Chrapusta S. J. Czarna M. Michalak M. Płaźnik A. (2019). Using anticipatory and drug-evoked appetitive ultrasonic vocalization for monitoring the rewarding effect of amphetamine in a rat model of drug self-administration. *Behav. Brain Res.* 376:112187. 10.1016/j.bbr.2019.112187 31473284

[B37] Larsson K. (1956). *Conditioning and sexual behavior in the male albino rat.* Stockholm: Almqvist & Wiksell.

[B38] Larsson K. (1967). Testicular hormone and developmental changes in mating behavior of the male rat. *J. Comparat. Physiol. Psychol.* 63 223–230. 10.1037/h0024358 6050026

[B39] Ma S. T. Maier E. Y. Ahrens A. M. Schallert T. Duvauchelle C. L. (2010). Repeated intravenous cocaine experience: development and escalation of pre-drug anticipatory 50-kHz ultrasonic vocalizations in rats. *Behav. Brain Res.* 212 109–114. 10.1016/j.bbr.2010.04.001 20382187 PMC2873056

[B40] Mahler S. V. Moorman D. E. Feltenstein M. W. Cox B. M. Ogburn K. B. Bachar M. (2013). A rodent “self-report” measure of methamphetamine craving? Rat ultrasonic vocalizations during methamphetamine self-administration, extinction, and reinstatement. *Behav. Brain Res.* 236 78–89. 10.1016/j.bbr.2012.08.023 22940018 PMC3482304

[B41] Matsumoto J. Nishimaru H. Takamura Y. Urakawa S. Ono T. Nishijo H. (2016). Amygdalar Auditory Neurons Contribute to Self-Other Distinction during Ultrasonic Social Vocalization in Rats. *Front. Neurosci.* 10:399. 10.3389/fnins.2016.00399 27703429 PMC5028407

[B42] Mulvihill K. G. Brudzynski S. M. (2018). Non-pharmacological induction of rat 50 kHz ultrasonic vocalization: Social and non-social contexts differentially induce 50 kHz call subtypes. *Physiol. Behav.* 196 200–207. 10.1016/j.physbeh.2018.09.005 30201573

[B43] Nikiforuk A. Kos T. Fijał K. Hołuj M. Rafa D. Popik P. (2013). Effects of the Selective 5-HT7 Receptor Antagonist SB-269970 and Amisulpride on Ketamine-Induced Schizophrenia-like Deficits in Rats. *PLoS One* 8:e66695. 10.1371/journal.pone.0066695 23776692 PMC3679060

[B44] Panksepp J. (2005). Beyond a Joke: From Animal Laughter to Human Joy? *Science* 308 62–63. 10.1126/science.1112066 15802592

[B45] Pfaff D.(ed.) (2017). “Central nervous system arousal fueling instinctive behaviors,” in *How the Vertebrate Brain Regulates Behavior*, (Cambridge: Harvard University Press), 215–226. 10.4159/9780674978751-009

[B46] Reinhold A. S. Sanguinetti-Scheck J. I. Hartmann K. Brecht M. (2019). Behavioral and neural correlates of hide-and-seek in rats. *Science* 365 1180–1183. 10.1126/science.aax4705 31515395

[B47] Sales G. D. (1972). Ultrasound and aggressive behaviour in rats and other small mammals. *Anim. Behav.* 20 88–100. 10.1016/S0003-3472(72)80177-5 4677167

[B48] Sales G. D. Pye J. D. (1974). *Ultrasonic communication by animals.* London: Chapman and Hall.

[B49] Simola N. (2015). Rat Ultrasonic Vocalizations and Behavioral Neuropharmacology: From the Screening of Drugs to the Study of Disease. *Curr. Neuropharmacol.* 13 164–179. 10.2174/1570159x13999150318113800 26411760 PMC4598429

[B50] Simola N. Costa G. (2018). Emission of categorized 50-kHz ultrasonic vocalizations in rats repeatedly treated with amphetamine or apomorphine: Possible relevance to drug-induced modifications in the emotional state. *Behav. Brain Res.* 347 88–98. 10.1016/j.bbr.2018.02.041 29505802

[B51] Simola N. Granon S. (2019). Ultrasonic vocalizations as a tool in studying emotional states in rodent models of social behavior and brain disease. *Neuropharmacology* 159:107420. 10.1016/j.neuropharm.2018.11.008 30445100

[B52] Taracha E. Hamed A. Krza̧ścik P. Lehner M. Skórzewska A. Płaźnik A. (2012). Inter-individual diversity and intra-individual stability of amphetamine-induced sensitization of frequency-modulated 50-kHz vocalization in Sprague-Dawley rats. *Psychopharmacology* 222 619–632. 10.1007/s00213-012-2658-4 22354555 PMC3402670

[B53] Tenk C. M. Wilson H. Zhang Q. Pitchers K. K. Coolen L. M. (2009). Sexual reward in male rats: Effects of sexual experience on conditioned place preferences associated with ejaculation and intromissions. *Hormones Behav.* 55 93–97. 10.1016/j.yhbeh.2008.08.012 18835271 PMC2659494

[B54] Wendler E. de Souza C. P. Dornellas A. P. S. Santos L. E. Ferreira S. T. Galduróz J. C. F. (2019). Mania-like elevated mood in rats: Enhanced 50-kHz ultrasonic vocalizations after sleep deprivation. *Prog. Neuropsychophar. Biol. Psychiatry* 88 142–150. 10.1016/j.pnpbp.2018.07.002 29981775

[B55] Wöhr M. (2021). Measuring mania-like elevated mood through amphetamine-induced 50-kHz ultrasonic vocalizations in rats. *Br. J. Pharmacol.* [Preprint]. 10.1111/bph.15487 33830495

[B56] Wright J. M. Dobosiewicz M. R. S. Clarke P. B. S. (2012). α- and β-Adrenergic Receptors Differentially Modulate the Emission of Spontaneous and Amphetamine-Induced 50-kHz Ultrasonic Vocalizations in Adult Rats. *Neuropsychopharmacology* 37 808–821. 10.1038/npp.2011.258 22030713 PMC3260979

[B57] Wright J. M. Dobosiewicz M. R. S. Clarke P. B. S. (2013). The role of dopaminergic transmission through D1-like and D2-like receptors in amphetamine-induced rat ultrasonic vocalizations. *Psychopharmacology* 225 853–868. 10.1007/s00213-012-2871-1 23052567

[B58] Wright J. M. Gourdon J. C. Clarke P. B. (2010). Identification of multiple call categories within the rich repertoire of adult rat 50-kHz ultrasonic vocalizations: effects of amphetamine and social context. *Psychopharmacology* 211 1–13. 10.1007/s00213-010-1859-y 20443111

[B59] Zala S. M. Nicolakis D. Marconi M. A. Noll A. Ruf T. Balazs P. (2020). Primed to vocalize: Wild-derived male house mice increase vocalization rate and diversity after a previous encounter with a female. *PLoS One* 15:e0242959. 10.1371/journal.pone.0242959 33296411 PMC7725367

